# Testing for Questionable Research Practices in a Meta-Analysis: An Example from Experimental Parapsychology

**DOI:** 10.1371/journal.pone.0153049

**Published:** 2016-05-04

**Authors:** Dick J. Bierman, James P. Spottiswoode, Aron Bijl

**Affiliations:** 1 Experimental Psychology, University of Groningen, Groningen, The Netherlands; 2 Brain & Cognition, University of Amsterdam, Amsterdam, The Netherlands; 3 LFR, Palo Alto, California, United States of America; 4 Brain & Cognition, University of Amsterdam, Amsterdam, The Netherlands; University of Hertfordshire, UNITED KINGDOM

## Abstract

We describe a method of quantifying the effect of Questionable Research Practices (QRPs) on the results of meta-analyses. As an example we simulated a meta-analysis of a controversial telepathy protocol to assess the extent to which these experimental results could be explained by QRPs. Our simulations used the same numbers of studies and trials as the original meta-analysis and the frequencies with which various QRPs were applied in the simulated experiments were based on surveys of experimental psychologists. Results of both the meta-analysis and simulations were characterized by 4 metrics, two describing the trial and mean experiment hit rates (HR) of around 31%, where 25% is expected by chance, one the correlation between sample-size and hit-rate, and one the complete P-value distribution of the database. A genetic algorithm optimized the parameters describing the QRPs, and the fitness of the simulated meta-analysis was defined as the sum of the squares of Z-scores for the 4 metrics. Assuming no anomalous effect a good fit to the empirical meta-analysis was found only by using QRPs with unrealistic parameter-values. Restricting the parameter space to ranges observed in studies of QRP occurrence, under the untested assumption that parapsychologists use comparable QRPs, the fit to the published Ganzfeld meta-analysis with no anomalous effect was poor. We allowed for a real anomalous effect, be it unidentified QRPs or a paranormal effect, where the HR ranged from 25% (chance) to 31%. With an anomalous HR of 27% the fitness became *F* = 1.8 (*p* = 0.47 where *F* = 0 is a perfect fit). We conclude that the very significant probability cited by the Ganzfeld meta-analysis is likely inflated by QRPs, though results are still significant (*p* = 0.003) with QRPs. Our study demonstrates that quantitative simulations of QRPs can assess their impact. Since meta-analyses in general might be polluted by QRPs, this method has wide applicability outside the domain of experimental parapsychology.

## Introduction

Recently it has become clear that experimental research, most notably in social psychology and cognitive neuroscience, but also in the medical literature, is plagued by questionable research practices (QRPs). In 2012 John, Loewenstein & Prelec [1] (hereafter referred to as JLP) surveyed 2,000 psychologists and determined the incidence of QRPs such as post hoc selection of studies to publish resulting in a file drawer, or having multiple dependent variables, hypotheses, statistical techniques, stopping rules, data-transformations (like outlier treatments) or conditions. QRPs may result in inflated P-values and Ioannidis [2] has suggested that some published research findings are actually incorrect due to QRPs, which bias the findings. According to Simmons et al. [3], undisclosed flexibility in data collection and analysis makes it theoretically possible to present too many datasets as ‘significant’. They reported simulations of these practices substantiating this claim. In 2015 the replication rates in psychology were assessed in a collaborative project [4]. While 97% of the original 100 studies were statistically significant, only 36% of the 100 replications were. No explanation is given for the failures to replicate but no signs of deception or methodological errors were found in the original reports. QRPs are seldom reported of course.

Meta-analyses (MAs) are sensitive to the accumulation of small, systematic errors but by quantitatively evaluating how much inflation can occur due to QRPs, we can estimate its impact on MAs. In the past the primary QRP that has been investigated was publication bias and the resulting file drawer. In this article we extend this to analyzing quantitatively the potential contribution of publication bias together with other QRPs.

We consider QRPs in the context of a meta-analysis database of Ganzfeld–telepathy experiments from the field of experimental parapsychology. The Ganzfeld database is particularly suitable for this study, because the parapsychological phenomenon it investigates is widely believed to be nonexistent. On the assumption that it is, the dataset would be a good example of how purely random data can be distorted into the appearance of significance.

In the following paragraph we present a synopsis of the paper. First we describe the field in general and then the actual protocol used to measure telepathic performance. Then we describe the meta-analytic database that is the result of more than 100 Ganzfeld-telepathy experiments. We will discuss why we have restricted ourselves to the period after 1985, thereby reducing the dataset to ~80 experiments. After presenting all potential QRPs for this specific protocol, including of course publication bias, we simulate these QRPs one by one showing what the effects are if *every* researcher has engaged in this particular practice whenever possible. Finally we will combine all QRPs in one simulation but rather than an unrealistic 100% prevalence we will allow the prevalence to vary in an interval around the values reported in the literature. The prevalence used here is the probability that a researcher will engage in a specific QRP whenever this is possible. If the prevalence for the QRP of rounding off p-values in the interval 0.05–0.06 is 100% this can only happen in ~1% of the cases. We distinguish this use of the word prevalence from admission rate, which is defined as the proportion of researchers who admit to using a particular QRP at least once.

Each simulation will produce a fit with the empirical database. We will use a genetic algorithm to find the prevalence and other QRP-parameters that produce the best fit. In sum the goal of this article is to demonstrate how QRPs can be simulated in a MA and to estimate the quantitative effect of QRPs on MA results.

### Experimental Parapsychology

Experimental parapsychology uses generally accepted scientific methods to study alleged anomalous phenomena such as telepathy, clairvoyance, precognition and psychokinesis. The field is relatively small, partly because there is little funding available for parapsychological research and also because publishing on these controversial topics is said to be detrimental to a scientific career [5]. The publications generated by serious parapsychological researchers during the last 150 years correspond to only about 3 months of research by current experimental psychologists [6]. Nonetheless, researchers have amassed large databases of experimental outcomes for each of the anomalous phenomena mentioned above, and each of these databases, taken at face value, strongly suggests the existence of an anomaly. Most proponents of parapsychology claim that these meta-analytic results are the ‘best evidence’ for paranormal phenomena [7, 8].

### The Ganzfeld telepathy experiment

In a Ganzfeld (GF) trial the subject (the ‘receiver’) is exposed to white noise and a homogeneous non-patterned red light, and reports his/her experiences, while at a distant location somebody (the ‘sender’) attempts to ‘send’ information relating to a randomly selected target (a picture or movie clip). At the end of the experiment the subject has to select an unhandled copy of the actual target from a set of 4 images or clips (1 target + 3 decoys). When the subject selects the target this is called a ‘hit’. Each subject generally contributes one data point, a ‘hit’ or a ‘miss’. Simulation of a GF experiment using this protocol is straightforward. The Mean Chance Expectation (MCE) hit rate (HR) is 25%. The average GF study reports a HR around 31% rather than the expected 25%.

### The Ganzfeld database

GF experiments date from the 1970s. In 1985 Honorton [9] claimed that the 28 studies published to that date with a mean HR of 35% constituted strong evidence for a real anomaly. For a detailed description of the history of the database see the supplementary materials. The most complete published meta-analysis of GF experiments today pools these 28 studies before 1985 with the 80 studies published since 1985 [10]. Storm et al removed 6 outliers (3 at the high HR side and 3 on the low HR side) in order to produce a homogeneous dataset. They were left with 102 studies, 24 are from the pre-1985 period and the remaining 78 are from the period from between 1985 and 2010. Storm et al. [10] report the overall meta-analytic results as follows:

“…The homogeneous database consists of 102 studies: mean *z =* 0.81 (*SD* = 1.23; range: 2.30 to 4.32), … and Stouffer *Z* = 8.13 (*p* = 10^−16^). … With Rosenthal’s …. file drawer formula, there would have to be approximately 2,414 unpublished and non-significant papers in existence to reduce our significant Stouffer *Z* to chance…”.

Note the extremely small P-value of *p* ~ 10^−16^. This result has been criticized by Hyman [11] noting that the pre-1985 database contained too many obvious flaws and that it wasn’t allowed anyway to combine databases that were so different.

Note also the rather old-fashioned method used to quantify the file drawer. We will return to the file drawer later in a separate section describing all QRPs. Because some of the pre-1985 studies had procedural weaknesses that are impossible to simulate, we decided to use the post-1985 studies (see S1 File for the inclusion/exclusion argument). The goal of the current study is to determine how much of this apparently significant result may be explained by QRPs

### Characterization of the Meta-Analytic Database

In order to fit the simulated experiments to the meta-analytic results we will characterize the dataset by a number of quantitative aspects, such as the HR. Discrepancies between these quantitative aspects and the same quantities derived from the simulations will be used to assess how closely the simulations match the empirical MA data. The final GF database used in the simulations is available in S1 Dataset.

### Sample size distribution

**Fig 1:**
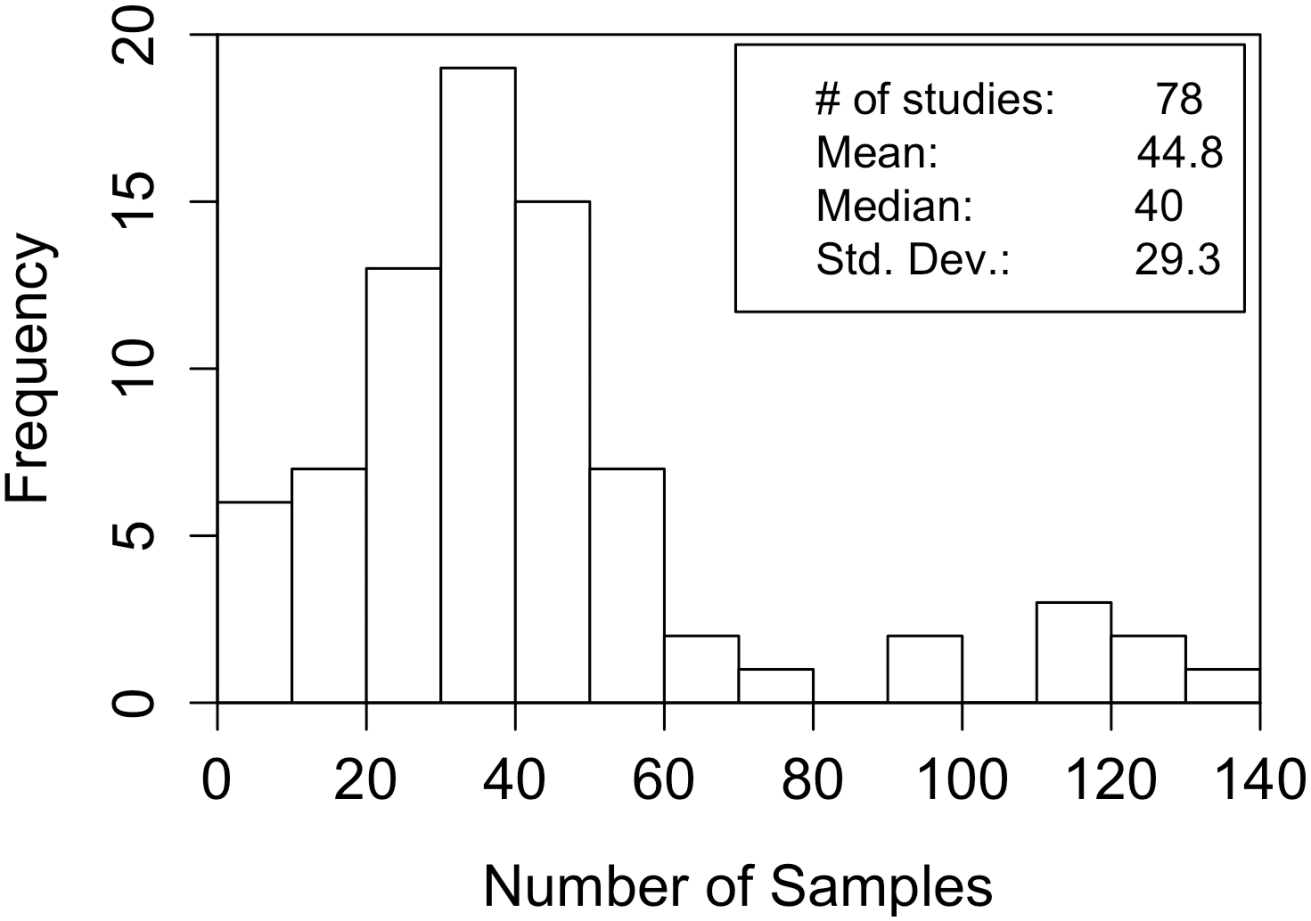
Distribution of Sample Sizes (= number of samples) in the database.

Fig 1 shows the actual distribution of sample sizes in the resulting GF database. The mean number of participants (and hence data points) in the GF studies in this database was 44.8. The effect of some QRPs is arguably dependent on the sample size, so we use the actual distribution of experimental sample sizes in our simulations.

### Hit Rates vs Sample Size

The hit rate of a specific study is the number of hits divided by the number of trials for that specific study. The mean HR (mHR) of the 78 studies is 31.15% If the raw data of all experiments are pooled there are in total 1083 hits in 3494 trials, a total HR of 30.99%. This difference between mHR and HR is due to the small negative correlation between HR and number of trials (N).

**Fig 2:**
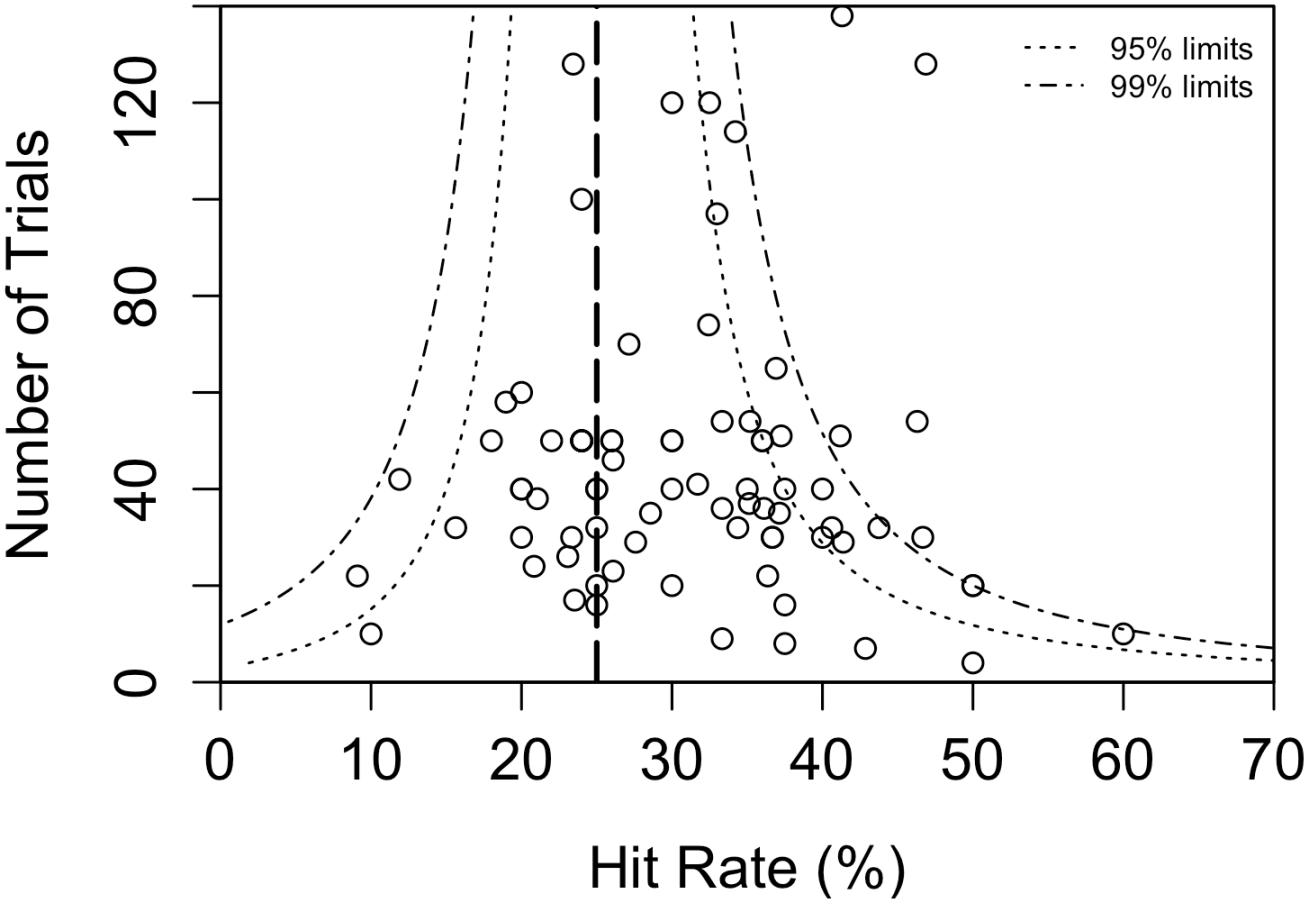
Funnel plot of hit rates in the database.

In Fig 2 we show the studies with their sample size and hit rate. This plot shows that non significant GF studies have been published and that there are a few outliers. The largest outlier concerns a study with a population selected from an Art school [12]. Due to the outliers, a regression analyses to estimate the file drawer is not allowed. But it even doesn’t make any sense to use deviations from expected P-value distributions because not only the QRP of publication bias creates distortions in the expected P-value distributions. Actually *each* of the QRP’s does that, as can be seen from the fitting values for fitting with the observed empirical P-value distribution in table sup. Therefore none of the existing methods such as PET-PEESE or Pcurve analysis, can be used to estimate the file drawer in our analysis. Actually all these techniques are only useful to estimate the file drawer under the condition that there are no other QRPs which affect the P-value distribution.

Note that HR can be employed as an effect size (ES) because it is independent of *N*. The Cohen’s *d* ES is defined as *d* = *Z / √N* and *Z* is given by:

$$Z\;=\;\frac{N(HR-0.25)}{\sqrt{N\times 0.25\times 0.75}}$$

From this formula the (linear) relation between ES and HR follows:

ES ≅2.31(HR-0.25)

Cohen’s effect size of this database is *d* = 0.138. In the simulations and further presentation of results we will use HR’s throughout.

### Correlation between Hit Rate and Sample Size

In principle HR should be independent of sample size, because each subject is tested independently. In the current database the non-parametric (Spearman’s rho) correlation between these two variables is -0.112. This is a quite small and non-significant relation. However we feel justified in using this correlation as one of the fitting parameters because in the total GF database of 102 studies the negative correlation is -0.206 (*p* = 0.038, two-tailed). Furthermore these negative correlations have been found in many meta-analyses within parapsychology as well as within psychology in general, so we conclude that this is a real aspect of the data [13]. Note that by removing the part of the database that contains the less rigorous pre-1985 studies the negative correlation has decreased in magnitude. This suggests that the negative correlation may be associated with the use of QRPs.

We will require our simulations to replicate not only the empirical HRs but also the internal effects like the negative correlation between HR and sample size and the peculiar P-value distribution.

### P-value distribution

**Fig 3:**
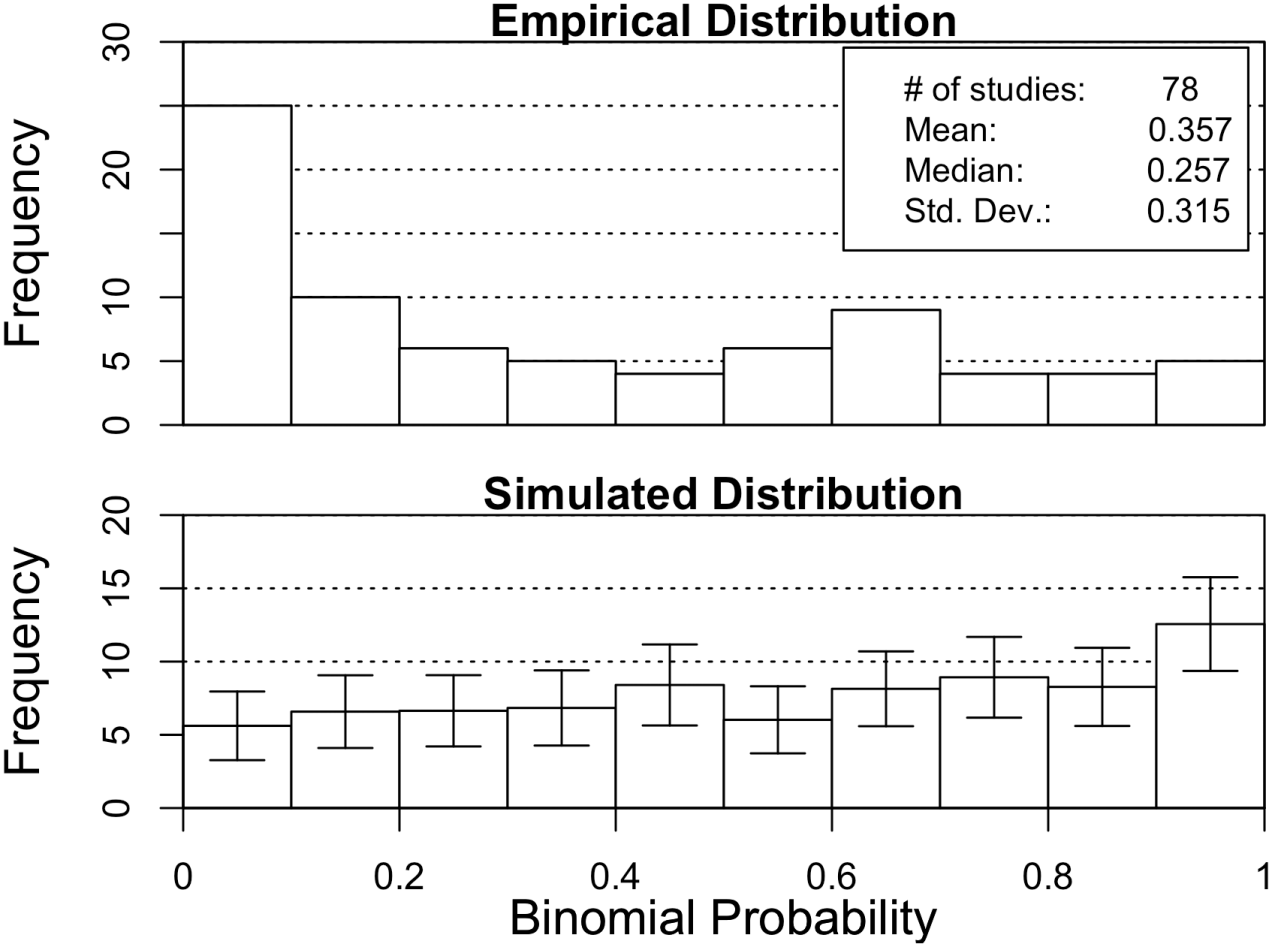
Distribution of binomial P-values. Upper pane: the experimental GF database. Lower pane: Simulated Null-distribution.

The binomial P-value distribution (Fig 3) has many more significant studies than would be expected by chance. The empirical P-value distribution is significantly different from the chance null distribution (chi-square = 25.4, *df* = 9, *p* < 0.003). A closer look at the empirical distribution indicates that the intervals with the largest differences from the null distribution are 0 < *p* < 0.1 and .7 < *p* < 1.

### Questionable Research Practices

There are a few known QRPs mentioned by JLP, such as ‘failing to report all dependent variables and conditions’ that are not applicable in the post-1985 database because of the specific GF research paradigm and its standardization. Apart from a number of non-applicable QRPs we identified two hitherto not explicitly described QRPs that we abbreviated: Confirmation-to-Pilot (CtoP) and Pilot-to-Confirmation (PtoC).

For each of the applicable questionable research practices there are two or three parameters (see table a in S1 File). For each QRP there is a prevalence. There are many reasons why researchers may differ in opinion as to what reasonable values for these prevalences are and these might differ between various populations of experimental researchers. Therefore, before giving the descriptions of the QRPs we will first describe the way we determined the reasonable prevalence intervals.

### Prevalences

The JLP study was the first to measure how many researchers have used specific questionable practices at least one time in their career. In order to promote truthful answers, JLP used a Bayesian truth serum (BTS) scoring algorithm developed by Prelec [14], which uses respondents’ personal answers, and their estimates of the sample distribution of answers, as inputs into a truth-rewarding scoring formula. The more truthful a respondent was scored, the larger the donation made to a charity of the respondents’ choice.

When respondents answered the question regarding their own use of QRPs, this was called the ‘admittance rate’. Responses having to do with their colleagues’ use of QRPs was called ‘prevalence’. Fiedler & Schwarz (FS) [15] argue that admittance figures must not be confused with prevalences (defined as the frequency of occurrence) because a researcher who admits committing a practice at least once might in fact have done it frequently. Problematically, the interpretations of the JLP results have not always made a clear distinction between admission rate and prevalence. FS ran their own replication and found slightly lower admittance figures. If we take into account that their respondents were likely sensitized due to the earlier published JLP paper these findings basically confirmed the JLP findings. FS also measured repetition rates. They not only asked respondents if they *ever* engaged in this practice’ but also how *often* they engaged in this practice. Combination of these two figures did result in prevalence figures that are much lower than the admittance figures of JLP and FS. However, from the wording of their question about repetition rates, it is clear that FS did not note the complication we noted earlier that not every QRP can possibly be applied in every study. For instance their repetition rate for the rounding off error is 17% (their Fig 2) that, together with the admission rate, results in a frequency of this particular QRP of 3.4% even though the number of times this specific QRP can be applied is around 1%. It would not be surprising if an experimenter who admits to having committed the p-value rounding off QRP once, would always use this practice in a similar situation. In our simulations we would give this experimenter a prevalence of 100%. This experimenter is inclined with a specific probability, in this case of 100%, to use the QRP in every case that allows for it. It is this probability that we use in our simulations and which is only approximated in the JLP and FS studies. In our simulation we therefore do not commit ourselves to a specific value of the prevalence parameter but instead use an interval of prevalences around JLPs BTS prevalence figure for each specific QRP if we have no other information. However, sometimes there is additional information. For instance, when trying to simulate the effect of fraud we used the prevalence figure from JLP but we also used information about detected fraud in the field of experimental parapsychology. These two sources of information provided converging figures giving greater confidence in the estimate by JLP.

With regard to optional stopping, a glance at the database shows sample sizes ranging from 5 to more than 100. About 45% of the sample sizes seem to be set in advance (*N* = 10, 20, 30 etc. with frequencies around 10) but the other sample sizes are unusual and appear only one or two times. No explicit statements in the publications such as ‘using a power-analysis to determine sample size’ are available. Therefore we might suspect that the QRP of not specifying sample size in advance and hence giving the freedom to use practices like optional stopping and optional extension, has been used. JLP estimated the prevalence of these particular QRPs to be 58% for collecting more data than planned and 22% for stopping prematurely if the results reach significance. These figures are in line with our estimate of ~40% based upon the publication of a round number of trials. These considerations therefore resulted in our choice of prevalence values (Table 1).

**Table 1:** Review of applicable QRPs and their prevalences.

Description	QRP	JLP (%)	Used Prevalence interval
Confirmation to Pilot	CtoP	40.7	20–50
Pilot to Confirmation	PtoC	-	20–50
Optional Stopping	OS	44.1	20–50
Optional Extension	OE	56.5	40–60
Publication Bias	PB	49.5	40–60
Biased removal of Ss	rmSs	35.1	30–55
Deception	Fraud	4.3	~3

Another line of criticism with regard to the choice of this reasonable prevalence interval is the possibility that these prevalences might differ between populations of researchers as we noted already with regard to the lower admission rates from the German population in the FS study. Parapsychologists may be more sensitized to these issues due to the strong scrutiny, to which their work is subjected [16]. Additionally, publication policy in the parapsychology field is such that it is possible to publish non-significant results, and a non-significant outcome is not a danger to the career of the parapsychologist. On the other hand, parapsychologists may be more driven by a non-materialist or spiritual worldview that they try to defend. The cautious approach therefore is to assume that the prevalence values of the use of QRPs in parapsychological research are similar to those measured in experimental psychology.

### QRPs which were simulated

Here we describe all the QRPs that we modeled and simulated.

#### Confirmation to Pilot (CtoP)

We identified a version of optional stopping that produces a file drawer that is generally not recognized. CtoP occurs when a confirmatory experiment is started but then halted after a few subjects (parameter: *Trialnr*) when results do not meet expectations, for instance if the observed HR is significantly lower than expected (parameter: *P-crit*). The experimenter then adjusts some aspect of the protocol and restarts the experiment discarding the data from the initial unsuccessful sessions. This data is in fact properly part of the file drawer, though it is frequently not treated as such because researchers mistakenly think the file drawer only contains finished but unpublished studies.

#### Pilot to Confirmation (PtoC)

This particular QRP has not been extensively discussed in the literature yet. Most labs and researchers, when starting a GF experiment, will run some pilot trials (parameter *N-trials*) to get experience with the equipment and the rather complex procedure. The intention is to check everything and, if no problems are encountered, to run a confirmatory experiment. If the pilot trials are technically successful (parameter: *pCrit*) and show a promising HR, then the experimenter may consider the pilot data as a part of the confirmatory experiment.

#### Optional stopping (OS)

Simulation of OS showed that it has no noticeable effect on the obtained HR (see results table a in S1 File). This was somewhat surprising since students are generally taught that this particular flexibility in experimental practice gives misleadingly inflated scores. Many articles have appeared on the effects of OS or, as it was also called, ‘repeated significance testing’ on accumulating data [15]. However OS does have an effect on the P-value distribution [17] and the correlation between sample size and effect size. Apart from the prevalence, there is one other parameter, the trial number (*trialnr*) at which repeated significance testing starts. The criterion to stop was always set to *p* < 0.05.

#### Optional Extension (OE)

This QRP, often considered to be a special form of OS, is probably the most well known QRP. If the experiment has not quite reached *p* < 0.05 (parameter: *P-crit*) a number of extra, unplanned, trials (parameter *extra-N*) are added in the hope that the final P-value will be less than 0.05.

#### Selectively reporting studies that have significant results: publication bias (PB)

This QRP occurs when experimental questions are addressed using several studies but only the study with significant results is published. In the GF database the research question for each experiment is identical; therefore in this context we can consider the file drawer as representing the unreported studies.

Post hoc selection of studies for publication, producing a file drawer of unpublished studies, has been extensively discussed in the literature. The usual way to treat this problem in parapsychology has been to calculate how many unpublished studies would be required to eliminate the reported effect using either fail-safe formula [17] or P-curve analysis [18]. For instance, Storm et al [10] calculated that 2,414 unpublished studies were required to eliminate the overall results of the GF database. Storm et al argue that this number, given the limited resources of the field and the acceptance of publishing negative findings, is unreasonably large. However Scargle [19] pointed out that researchers calculating this number generally assumed incorrectly that the decision not to publish is unbiased. He argues that assuming that the unpublished studies have zero effect size is incorrect.

Franco et al. [20] analyzed the file drawer effect in a group of experiments known as TESS (Time-sharing Experiments in the Social Sciences). TESS is known for its quality and consists of a known population of studies with full accounting of both published and unpublished ones. They classified the studies both on results (null, mixed & strong results) and on publication status. Of the total number of 221 studies investigated about half were published. Only 20% of those with null results were published, while studies with strong and mixed results had publication rates of 60% and 50% respectively

A search by Blackmore of unpublished GF experiments in 1980 yielded 19 yet unpublished versus, at the time, 31 published studies with no significant difference in outcome measures [21]. She therefore concluded: “The bias introduced by selective reporting of ESP GF studies is not a major contributor to the overall proportion of significant results.”

A survey of all finished studies at the Koestler Parapsychology Unit of the University of Edinburgh revealed that 15% of the non-significant and 70% of the significant studies were reported [22]. These figures confirm the publication bias observed in the TESS studies. Thus, in both fields there was a strong bias: the probability of publication increased by about 40%-55% when comparing strong results to null ones. When TESS researchers were asked why they didn’t publish null results, 15 out of the 26 respondents reported abandoning the project because of the low publication potential (even if they found these results interesting.) A smaller group (9) reported decreasing the priority of writing up null results, in favor of other projects. We will use a publication probability function derived from the empirical findings of Franco et al. To avoid using a likely unrealistic discontinuous PB function, we fitted the Franco step function with a continuous function:

$$pubprob\;=\;\frac{25+40(tanh(2-10p)+1)}{100}$$

where *pubprob* is the probability that the study will be published and *p* is the P-value of the study. This QRP has no free parameters (except of course the prevalence for that practice).

#### Deciding to exclude data post hoc (RmSs)

As formulated, this QRP appears basically fraudulent. However JLP report a defensibility rating of 1.61 (0 = indefensible, 1 = possibly defensible, 2 = defensible). Most probably, respondents were thinking about removal of outliers, not of subjects. In the case of GF research there can be no outliers, as there is only one binary data point per subject. In a lot of psychological research it is common practice (but still questionable) to remove subjects on the basis of reasons that weren’t specified *fully* in advance. For example, if a subject falls asleep during an experimental test, complains extensively about the temperature, or is late, researchers could argue that this subject should be removed. The problem is that if such a subject has a result that conforms to the desired hypothesis, the experimenter may be non-consciously less inclined to remove the subject. If subject removal happens blindly before inspection of the data, then the practice will not introduce a bias. However in GF research experimenters are generally not blind to the outcome of a session.

This QRP was modeled by its prevalence and by the percentage of subjects that was removed. The minimum of removed subjects was set to one (if the QRP occurred at all in the simulation as determined by a random decision based on its prevalence). The idea is that even in small studies where a fixed percentage would result in a number below 1, an experimenter generally can find a situation that ‘justifies’ removal of one subject.

Removing a larger percentage than 5% of subjects with misses (parameter ‘% of N’) will make the *post hoc* arguments for removal that an experimenter has to come up with increasingly artificial and will basically turn this practice into fraud (see ‘Fraud’). This QRP is akin to the asymmetric behavior of experimenters with respect to experiments that are close to *p* = 0.05. If *p* is slightly over 5% they will check their data and methods but when *p* is slightly smaller than 0.05 they might be less inclined to do so.

#### Fraud

Surprisingly about 4.4% of the respondents in the JLP study admitted to fraud. However it is difficult to assess whether this percentage also holds for researchers of paranormal phenomena. In the past 50 years, three high profile fraud cases by parapsychologists have been reported [23]. Two of these cases have been the subject of claims and counter-claims and the exact truth is impossible to determine. All these cases concerned researchers known for their long years of work in the field and who had published numerous papers. The total number of psi researchers with similar publishing records is estimated to be 200. From these numbers it seems that the percentages of fraudulent researchers in the field of experimental psychology and of experimental parapsychology are of the same order of magnitude. It is impossible to simulate this practice, so we had to correct the database by removing studies before we ran the simulations. We decided on the basis of the arguments given above that one senior researcher in the 80 studies post-1985 database might be guilty of deception. In order to take into account the contribution of deceptive research to the database we thus removed the two studies of one senior researcher, the person who had been implicated in errors in the randomization procedures. These studies were quite significant with HRs of 35% and 41%. In the database there are 29 principal investigators, so removal of one of them (3.4%) is near identical to the prevalence of deception found by JLP (4.4%).

## Method

### Simulation of QRPs

Simulation software was written in Real Basic (2011, Release 4.3), and developed independently in the statistical programming language R [24]. The full annotated R-source of the simulation software is in S1 Software. Each trial in a simulated experiment had the probability of a hit preset to 25% when simulating no anomalous GF effect or unknown QRP. Simulations generally proceeded in 300–500 sets of 78 experiments for a total of ~30,000 simulated experiments per run. The sample sizes for the simulated experiments were selected from the actual distribution of sample sizes in the database in such a way that after one set all sample sizes of the meta-analysis had been used. In such a simulated experiment, the QRPs were applied probabilistically. For each QRP there was a prevalence figure representing the probability that an experimenter would be ‘using’ this particular QRP. Then in each simulated experiment a random decision was taken whether to apply each QRP with the probability equal to that QRPs’ prevalence. This can be conceptualized as a simulation of the experimenter followed by a simulation of the experiment run by this experimenter. After the simulation of one set of 78 experiments, the 4 quantitative outcome measures are calculated to compare with the empirical database. Because we repeat this process 300–500 times we can calculate the standard deviations of the outcome measures, with the set (that is the simulated MA) as the unit of analysis. Software was validated by comparing the outcomes of the simulations written in *RealBasic* and the simulations independently written in *R*.

### Finding the optimal fit with the empirical data

Associated with each QRP is the prevalence, indicating the probability that researchers will use the QRP if the situation arises, and some free parameters. These were described in the section ‘description of QRPs’. These parameters are considered free parameters when seeking a simulation that produces results that best fit to the empirical data. This is an example of an optimization problem where we sought the maximum, or minimum, of some function. In this case we wish to find those values of the QRP parameters that result in a simulation of the meta-analysis which most closely resembles the empirical results. To this end, we defined a fitness parameter which is small when the simulated and empirical results are close and increases as the results differ more. The fitting itself, searching the QRP parameters that result in a simulated meta-analysis closest to the empirical meta-analysis, was performed by a Genetic Algorithm (GA) [25, 26].

GAs are a widely used method of solving optimization problems that is the problem of finding the maximum, or minimum, of a function, usually called the objective function, over a given domain of its independent variables. GAs have the advantage over analytic hill-climbing algorithms of not finding suboptimal solutions when the optimization landscape is complex and has many maxima (or minima). A more detailed explanation of the GA method is given in the supplementary materials.

In our case the objective function, Fitness (*F*), was defined as the sum of the squares of Z-scores that combined the normalized deviations of four metrics comparing the simulated meta-analyses to the actual one. Since this function increases when simulations differ more from the empirical MA, the GA was configured to find the minimum value of the objective function. Two of these quantities were derived from the HR by calculating the HR per study and averaging those to a mean HR (*mHR*) and the HR over all 3,494 trials comprising the 78 experiments (HR). These two HR measures were different and we computed the mean (*μHR*) and difference (*ΔHR*) of them. These two quantities were converted to Z-scores using the means and standard deviations obtained from our simulations. The remaining two Z-scores that went into the objective function were the *Z* transformed sample-size vs effect-size Spearman’s rho correlation (*Z*_*ρ*_) and the Z-score (*Z*_*Χ*_) derived from the P-value distribution fit (expressed as a Pearson’s chi-square transformed into a Z-score). That is:

$$Fitness\;=\;Z_{\mu HR}^{2}+\;Z_{\Delta HR}^{2}+\;Z_{\rho }^{2}+\;Z_{\chi }^{2}$$

where,

$$Z_{\mu HR}\;=\;\frac{\overset{-}{{\mu HR}_{Sim}}-\;{\mu HR}_{MA}}{std({\mu HR}_{Sim})}$$

$$Z_{\Delta HR}\;=\;\frac{\overset{-}{{\Delta HR}_{Sim}}-\;{\Delta HR}_{MA}}{std({\Delta HR}_{Sim})}$$

$$Z_{\rho }\;=\;\frac{\overset{-}{\rho_{Sim}}-\;\rho_{MA}}{std(\rho_{Sim})}$$

$$Z_{\chi }\;=\;\overset{-}{ZOP(\chi^{2}(D_{Sim},D_{MA}))}$$

and where overbar denotes taking the mean, *std* the standard deviation, and *χ*^*2*^ is the chi-square test whose arguments are the distributions of counts *D*_*1*_ and *D*_*2*_ and which returns the probability of the resulting chi-square value and *ZOP(P)* is a function that returns the Z-score derived from the probability *P*.

It should be noted that *Z*_*ρ*_ and *Z*_*Χ*_ are correlated and that this implies that the number of degrees of freedom required to calculate P-values from the fitness value is reduced. We therefore calculated P-values on the basis of Monte-Carlo simulations of the 4 component sum-of-squares where 2 of the components were forced to correlate in the simulation.

The Genetic Algorithm was run using a population size of 60 chromosomes until for 20 consecutive generations there was no further decrease in the fitness function. Each chromosome was a binary representation of one possible set of values of the 14 parameters that describe the modeled QRPs and a 15^th^ parameter for the assumed anomalous HR. The algorithm evaluated the fitness of each chromosome by decoding it into a set of 15 values for these parameters then running 200 Monte-Carlo simulations of the meta-analysis using those values.

## Results

### The simulated effect of each QRP in isolation

First we simulated all the applicable QRPs in isolation assuming that there was no anomalous HR effect and that every experimenter intended to use the practice whenever possible. Note that this assumed 100% prevalence does not imply that experimenters *always* do use that particular practice because, for many practices the data must be in a certain condition for the QRP to be applied. For example, optional stopping can only be applied if the running P-value becomes smaller than 0.05.

Table 2 shows that by applying these QRP the fit of the experimental database with the simulated database becomes better with one exception. Applying Optional extension in the simulation resulted in a worse fit with the empirical data.

**Table 2:** 

QRP	Relative gain in fit*	Increase in Hit Rate over 25% (MCE)
CtoP	+73%	+2.8%
PtoC	+30%	+0.8%
OS	+35%	+0.9%
OE	-63%	-0.3%
PB	+88%	+3.5%
RmSS	+52%	+1.2%

*Relative gain in fit = (fit with QRP—fit with no QRP)/fit with no QRP.

The best fit to the empirical data is obtained by the Publication Bias QRP. The fit *F*, becomes *F* = 9.9 (*p* < 0.05) indicating that even with a 100% prevalence for the publication bias QRP the simulation still differs significantly from the empirical database. Further detailed results of this 100% prevalence simulation of the QRP’s in isolation are given in the supplementary materials.

### Combined QRPs with Unconstrained Parameters

In order to see if it is possible to get an acceptable fit of simulations with the empirical MA results when experimenters use several QRPs simultaneously we first ran the GA fitting procedure with no constraints on the prevalence figures. In these simulations all parameters of all the QRPs and all their interactions were explored in parallel. The results show that a reasonable fit of *F* = 2.01 is reached after 35 generations. However, a few of the QRP prevalence figures converge to very high values. For instance, the simulations imply that 93% of experimenters use the publication bias QRP and 77% of them removed 5% of the subjects. These prevalence figures are well above the prevalence figures from the literature and above what we would expect on estimates provided by research by Franco [20], Blackmore [21] and Watt [22].

### Combined QRPs with Constrained Parameters

As described in the method section, a Genetic Algorithm was used to search for the optimal fit in the QRP parameter space under the restriction that the value of parameters were not allowed to go outside the reasonable parameter intervals. It is important to note that we used a broad interval rather than a single point estimate of the prevalences. A single point estimate would have left a very large unexplored parameter space. But in our simulation we used broad intervals of QRP parameters. As in the unconstrained case all parameters of all the QRPs and all their interactions were explored in parallel.

**Fig 4:**
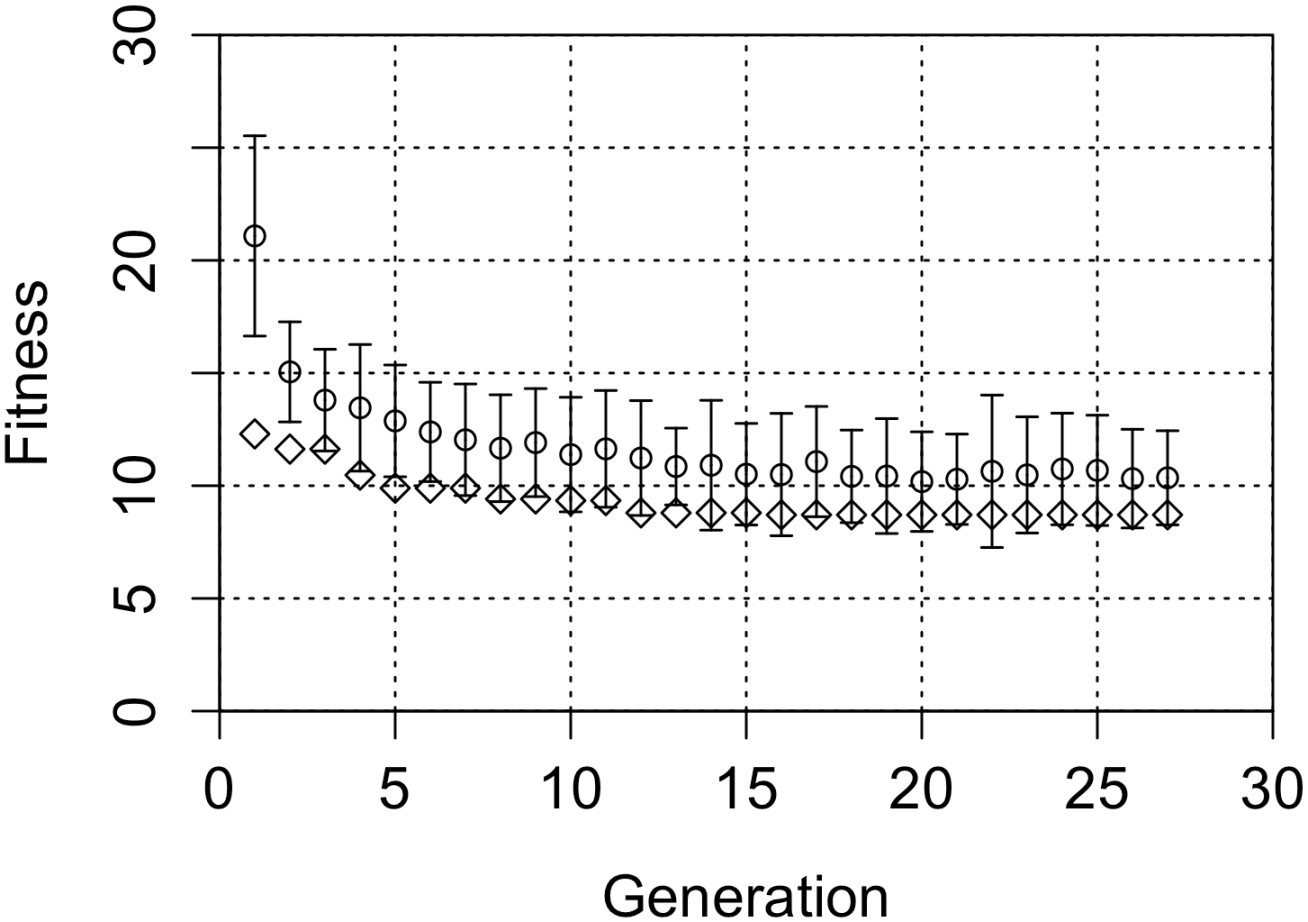
The fitting value as a function of generation of the Genetic Algorithm. The QRP-parameters are kept within a reasonable interval. Circular points show the mean fitness for each generation, with error bars of 1 SE, diamond points show the best fitness per generation.

It can be seen that the final best fitness value of 10.15 after reasonable application of combinations of QRPs is about the same as in the case of the unreasonable 100% prevalence file drawer effect (Fig 4). In this combined QRP analysis with restricted prevalence intervals, the prevalence parameter for publication bias converges to 58%. This effectively results in a realistic figure of 49.9% unpublished completed studies. The values of the other prevalences that are obtained and yield the optimal fit are as follows: C2P = 49%, P2C = 47%, OS = 32%, OE = 44%, PB = 58%, RmSS = 41%

The fit of 10.15 indicates that the simulated results still differ significantly from chance (*p* < 0.05). The main reason that the fit is still unsatisfactory is that the simulated HRs are about 2.5% too low. If we had restricted fitting to just the HR measures then the difference between the simulations and the empirical HRs would have been very significant (*Z* = -2.96, *p* < 0.01). The fit of the simulated and experimental P-value distribution, however, is very good (corresponding *Z* = 0.21) and also the correlation is reasonably simulated (simulation rho = -0.15 and experimental *rho* = -0.11, *Z* = 0.29)

The other QRP parameters that came out of this simulation were:

C2P is activated if *p* > 0.27 at trial 10 and P2C is activated if *p* < 0.29 at trial 7. OS starts checking if *p* < 0.05 at trial 23 and rmSS yields removal of 4.5% of subjects removed post hoc. Giving an average of 0.7 removed subject per study.

### Adding an anomalous component or an unknown QRP

All the simulations described so far have used a chance HR of 25%. With this restriction the simulations have failed to account for about 2.5% in the HR. We can also simulate either a real anomalous effect or an unknown QRP by allowing an excess hit rate over 25%.

**Fig 5:**
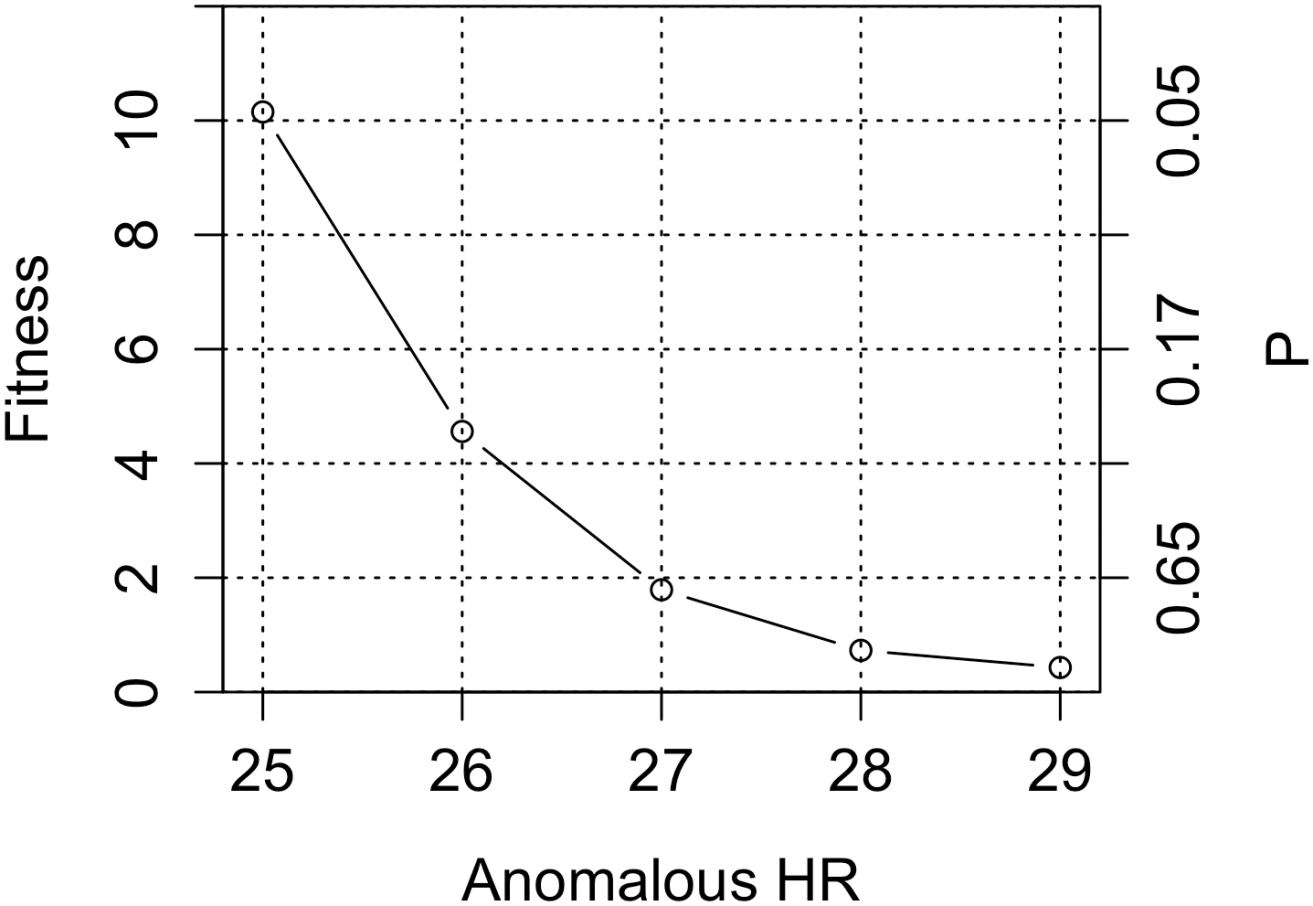
Fitness values when using reasonable QRP parameters and allowing for a small excess hit rate. The right Y-axis indicates the probability that simulations and experimental data are the same.

We therefore repeated the simulations with HRs of 26, 27, 28 and 29%. The fit improves with increasing anomalous HR (Fig 5). It can be seen that the effect of allowing excess hit rate is minimal after the hit rate becomes 27%. The fit, *F* = 1.79, for this case and the corresponding prevalences are all well in the reasonable interval.

## Discussion

The simulations of applicable QRPs in the controversial paradigm of GF that are presented in this article were intended to answer this quantitative question: How much of a meta-analytic result can be explained under the assumption that QRPs have been used to the same degree that has been reported in the general scientific literature? We have shown that the method of using several quantitative aspects of the MA database in a fitting procedure where we simulate these QRPs can answer this question. This method can be used to estimate the corrected effect size, that is the effect size which would be observed in the absence of all known QRPs. This is extremely important in order to be used in subsequent research especially if that subsequent research is focused upon answering how replicable the claimed effect is. In the case of GF research our simulations suggest an unexplained excess in HR of 2%. With this small effect, the required number of subjects to be tested for a probability of 80% to obtain a P-value of 0.05 is in the order of 700 subjects. In the history of GF experimentation, no single study has used this large a number of subjects due to the financial constraints of the field.

Splitting up this required number of subjects into smaller numbers for a coordinated parallel replication effort could be shown to be equivalent statistically to a single large study but only if QRPs like ‘Confirmation to Pilot’ or ‘Pilot to Confirmation’ can be excluded. These two QRPs relate to a single experiment and having many small experiments gives many options to use them while in a large experiment there is only one option, which considerably reduces the impact of these QRPs on the overall result. Actually these two QRPs could be the source of other observations in the psychological literature [13] of smaller effect sizes when using larger samples.

Baptista & Derekshani [27] found evidence that some subject populations, for instance artistic populations, have higher HRs than others. To obtain a reasonable power for GF studies, the only realistic option is to use special populations rather than using the average freshman psychology student. Comparing 11 free response telepathy studies using a selected population with 80 similar studies using a non-selected population showed that the effect size for the selected subjects was about three times larger than for the unselected subjects. For the GF database, the effect sizes uncorrected for QRPs for selected (artistic) population and the unselected population are *d* = 0.50 and *d* = 0.14 respectively. If we assume that studies with special subjects do not differ from studies with unselected subjects in terms of use of QRPs the estimated true effect size after correction for QRPs for the artistic population would be around 0.40 and a study with around 50 artists, like the Juilliard students used in the Schlitz-Honorton GF study [28], would have a power of 80% to establish this effect [29].

A consistent finding in the medical literature is that there are large discrepancies between results of meta-analyses and those of large scale randomized controlled trials [30]. The latter found for instance that meta-analyses would have resulted in the adoption of an ineffective treatment in 32% of the cases and in about the same percentage of the cases an effective treatment was rejected. Hence, a satisfactory power analysis result for each study has been suggested by Muncer as a required inclusion criterion in meta-analysis [31]. Muncer relaxes the power requirements for inclusion of a study in a meta-analysis to 0.50 on the basis of the weighted mean effect size of the initial database. In the case of the GF database only 6 studies, contributing a total of 748 trials would qualify on this criterion. Interestingly these would produce a mean HR of 31.2% (*p* < 10–4). But of course this result assumes that no QRPs were used in those 6 studies. If the impact of QRPs in these 6 studies was the same as in our overall analysis, their hit rate would be 27.1% (*p* = 0.07).

With regard to our simulation method there are a few issues to be discussed. The first issue is the selection of studies to be included in the GF database. We started with the largest database available, which included 108 experiments from 1974 to 2010, but decided to exclude studies before 1985 for reasons detailed in the Introduction. We should mention here that if we had used the total database including the pre-1985 studies we would have concluded that the percentage of the effect explained by our QRP’s would have been smaller. But that is consistent with the fact that the pre-1985 database arguably has more procedural weaknesses such as the possibility of sensory leakage, which is impossible to simulate.

We used the publication probability function reported in a study of psychologists in general. As noted, some have argued that the publication probabilities for studies in the field of experimental parapsychology could be totally different, and far more cautious, from those in general psychology research. For instance, some critics have argued that the publication probability function was such that only 1 in 20 studies had been published [32]. As a consequence, to produce the current database, about 2000 GF studies would have been required, from which about 1900 ended up in the file drawer. Given the costs in time as well as money of these studies, and the editorial policies in parapsychological journals which also publish non-significant studies, this seems to be unreasonable.

Another point of discussion is whether we really did discover and simulate all the QRPs that could be used in this type of experiments. Each of the QRPs used in our simulations (except those for optional extension and optional stopping) gave some 1% extra HR. This could suggest that, under the assumption that telepathy doesn’t exist, there are still 2 or 3 QRPs we failed to investigate. One that we did not implement is the QRP of manual data-entry. Nowadays the practice of hand scoring has largely disappeared due to the fact that most tests are done using a computer where the subject herself enters the responses or other data-items. Error rates are highly context specific [33] and range from 0.03% for experienced bank machine operators [34] to 1–2% for students performing a table lookup task [35]. The automated GF results, where data-entry was always directly into the computer, have however a slightly *larger* overall HR (30.77%) than the remaining studies where data-entry was generally less sophisticated (30.25%). Therefore we might conclude that manual data-entry probably didn’t introduce noticeable effects on the results.

It should be mentioned that the results of our simulations are not critically dependent on choices of the fixed point simulation parameters. For instance when simulating OS, we choose in our model for this QRP that the researcher starts contemplating early stopping and checking whether the p-value is smaller than 0.05 at trial 10. If we choose another trial number between say 8 and 20 where the experimenter starts checking the cumulative P-value, the simulation results are very similar. However there is one exception where a different choice would have had a larger impact: the fraction of subjects that an experimenter would remove from an experiment in a biased way. Again our choice here was based upon our experience in the field but some proponents of the psi-hypothesis might consider our assumed fraction too large.

Our exercises show that QRPs can account for large fractions of small effect size phenomena. What holds for this particular GF paradigm most probably also holds for small effect size of less controversial effects, especially in paradigms where the experimenters’ freedom is larger. Such is generally the case in most traditional paradigms. For instance, in GF experiments, unlike in many general psychology experiments, there are no outlier corrections and there is no freedom in preprocessing of physiological data.

The methodology used in our simulation and fitting procedure can also be applied to other meta-analytic databases. For each specific research protocol, one has to determine first what QRPs are possible. For instance one can easily simulate the effect of trying out different outlier correction procedures and picking the one that will give the ‘best’ results. The same holds for using different outcome measures. Other QRPs might be more difficult to simulate and therefore require careful examination of the original materials. This kind of quantitative evaluation of the possible contribution of QRPs to meta-analytic databases allows us to estimate the true effect size corrected for QRPs and therefore the required statistical power in further QRP-free experiments.

Given the assumptions we made about the reasonable intervals for the simulation parameters, we conclude that QRPs are capable of explaining about 60% of the effect size reported in the GF meta-analysis. Simulations allowing for excess hit rate whether due to a real anomaly or an as yet unnoticed QRP, confirmed this estimate.

## Supporting Information

S1 File Supporting Information. (DOCX)

S1 Software MA Simulation. (R)

S1 Dataset Ganzfeld Database. (XLS)
