# Supplementary material for: Testing for Questionable Research Practices in a Meta-Analysis: An Example from Experimental Parapsychology
Source: PLoS One. 2016 May 4;11(5):e0153049. doi: 10.1371/journal.pone.0153049 (PMC4856278; doi:10.1371/journal.pone.0153049)
Supplement: S1 File — (DOCX) [file pone.0153049.s001.docx]

# S1. Supplementary materials

### Submission:

### Testing for Questionable Research Practices in a Meta-Analysis: An Example from Experimental Parapsychology

## Discussion of meta-analytic dataset

### History of the database

Ganzfeld (GF) experiments started in the 1970s and in 1985 claims were made that the 28 of the 44 studies from which the same outcome measure, Hit Rate (HR), could be calculated had a mean HR of 35% and constituted strong evidence for an anomaly (1). This interpretation has been criticized on several grounds. Firstly, individual experiments have been criticized on grounds of the potential for sensory leakage and lack of effective control of normal explanations. Hyman (2) specifically presented several weaknesses in these data, including some practices that we today would label as Questionable Research Practices (QRPs). Most importantly, Hyman demonstrated that almost all studies used multiple outcome measures to test the major hypothesis: “Is there a significant deviation from chance?” In the early GF work different researchers had used 5 different indices: the direct HR, the binary HR, the sum of ranks, the normalized rating and a 10-bit binary scoring method. If the conservative Bonferroni correction had been applied, studies that reported a P-value of 0.05 actually should been considered to have a P-value of 0.25. However, these dependent measures used to test the same hypothesis are highly correlated and therefore the Bonferroni correction is over-conservative. In order to estimate the required true correction to the P-values, Hyman ran simulations of a GF experiment with a fixed number of trials. He calculated for each simulated experiment the p-values for the 5 different outcome measures and then choose the one that produced the most significant value. He concluded that when a P-value of 0.05 was claimed where this experimenter freedom was allowed, then in reality this P-value must have been close to P = 0.10. Honorton responded by evaluating the 28 studies (of the 44) where the HR was presented or could be inferred (9). He claimed that this analysis was now free of the QRP of using multiple indices and he reported still a highly significant combined (Stouffer) Z-score of 6.6 (p= 2.06 * 10^-11^). Honorton also responded to Hyman’s list of procedural weaknesses; for example, that about half of the studies failed to use duplicate target sets and hence sensory leakage through marks on the target picture could have occurred. As a result of the controversy between this skeptic and this parapsychologist, Hyman and Honorton came together and wrote a joint communiqué (3). They promoted pre-registration, and had some explicit recommendations for GF protocols. Following the communiqué, GF experiments were much more standardized, often automated and had hardly any procedural weaknesses and almost all used a single outcome measure (4). These studies form the core of the post-1985 database. But Honorton had started doing totally automated Ganzfeld-experiments even a few years earlier and the results thereof are also included in the post-1985 database.

### Justification for using the post-1985 dataset

While the pre-1985 dataset could be affected by the QRP of multiple outcome measures, this is not applicable to the post-1985 database. If that were the only difference we could have simulated the whole combined data set by allowing one extra QRP on the early studies. However the procedural weakness of sensory leakage caused by using a single target-set in the pre-1985 studies, is impossible to simulate but had apparently no effect on the results because the mean HR for the single target-set studies was smaller. However, Hyman also noted other procedural weaknesses such as ‘inadequate randomization’ and ‘inadequate security’, rejecting any study that used only one experimenter. In such studies there is a risk that the experimenter who is present at the judging is aware of the target or has a reasonable guess at it because (s)he is also involved in the target selection procedure. In the judging procedure at the end of a GF session, the experimenter generally plays an active role and can easily influence the subject’s choice in any direction. An observer who visited Eysenck’s parapsychology lab in 1980 and attended a number of GF sessions noted irregularities in the (very complex manual) randomization procedure that potentially would allow for one of the experimenters to get information about the target (5). The case was investigated by a committee appointed by the professional organization of parapsychologists, the Parapsychological Association (PA). The committee, consisting of PA members and a member of the professional Skeptical organization CSICOP, concluded that no indications of deception could be found. Their report however never became public, leaving an unwarranted taint (6).

Given the weaknesses and criticisms of the pre-1985 studies, we focused on the post-1985 dataset for our simulations. In that post-1985 dataset there were two contributions by the lab that had been accused of irregularities in the randomization procedure. We removed these two studies from the database. We justify this removal in detail in the main text paragraph on ‘fraud’. We included the studies that were removed by Storm et al (7) to obtain homogeneity, as these 6 studies were run after 1985 and this removal had only been done to enable statistical evaluation. Finally, we removed one study that used a judging procedure with 7 decoys rather than the standard 3 decoys. Thus our final data set included 78 studies which is publicly available and is supplied as supporting information.

## The simulated effect of each QRP in isolation

A review of the detailed simulation results of applying only one QRP at a time with a prevalence of 100% and with typical values for the QRP parameters is given in Table sup1a and b. In order to assess if application of the QRP results in a simulation that fits better or worse with the empirical data, we give the fit of the resulting mean MA simulation with the actual MA data as 4 Z-scores representing the deviations of the 4 key metrics, a) over-all HR, b) mean HR, c) Correlation between HR and sample size and d) the total p-value distribution. . The value of the overall fitting variable fit is the sum of the four Z^2^ scores. The fit is perfect when fit = 0.

**Table S1a. The effect of each of the applicable QRPs in isolation on the simulated variables.**

| **QRP** | **Fixed Parameters** | **Resulting data** | | |
| --- | --- | --- | --- | --- |
|  |  | **HR** | **mHR** | **Rho** |
| Database* |  | 30.99 | 31.1 | -0.11 |
| None** |  | 25.0 (0.74) | 25.01 (0.9) | 0.00 (0.12) |
| CtoP*** | P >.6 @ trialnr 10 | 27.5 (0.7) | 28.2 (0.9) | -0.16 (0.11) |
| PtoC*** | P <.4 @ trialnr 6 | 25.7 (0.7) | 25.8 (0.9) | -0.01 (0.1) |
| OS *** | P < .05 @ trialnr >15 | 25.0 (0.8) | 25.9 (1.0) | -0.12 (0.11) |
| OE **** | P < 0.2 then N= N+20 | 24.9 (0.6) | 24.6 (0.9) | +0.07 (0.11) |
| PB | Franco | 28.2 (0.8) | 28.8 (1.0) | -0.11 (0.11) |
| RmSS | 5% | 26.1 (0.8) | 26.3 (0.8) | -0.01 (0.11) |

**Table S1b. The effect of each of the applicable QRPs in isolation on Fit with each of the 4 metrics and the overall fit.**

| **QRP** | **Fit-values** | | | | |
| --- | --- | --- | --- | --- | --- |
|  | **HR** | **dHR** | **Rho** | **Pdistr** | **Overall** |
| None** | -7.8 | -0.2 | 1.16 | 4.52 | 83 |
| CtoP*** | -4.2 | 1.0 | -0.4 | 1.6 | 21.9 |
| PtoC*** | -7.0 | 0 | 0.91 | 2.78 | 57.7 |
| OS *** | -6.5 | 1.15 | -0.11 | 3.21 | 54 |
| OE **** | -8.1 | -0.89 | 2.05 | 8.06 | 13 |
| PB | -3 | 0.82 | 0.02 | 0.45 | 9.9 |
| RmSS | -5.83 | 0.03 | 0.89 | 2.21 | 39.7 |

* The ‘database’ entry gives the observed values in the empirical database.

** The ‘none’ entry gives the theoretical (25% HR, no QRP) fit to the database. The fit is the worst F=83.

*** P >.6 @ 10 means that this QRP is used when the running P-value is larger than 0.6 at trial 10.

**** The experiment is extended with 20 trials if P < 0.20 at the end of the planned experiment.

The table S1a shows that 4 of the 6 identified QRPs, when used in isolation every time they can be applied, produce an increased HR. Three of the QRPs in isolation, CtoP, OS and PB produce a substantial negative correlations between sample size and HRs similar to the correlation observed in the database. If we combine these QRPs, assuming a prevalence of 100% for each of them, this correlation becomes -0.58, much larger than the observed empirical value of -0.10. This suggests that in order to simulate a realistic negative correlation the prevalences of these 3 QRPs should be considerably reduced.

Unlike what is suggested in the literature (8), the simulation of optional extension of the experiment with extra subjects if the cumulated result at the planned N is just above 0.05 does not yield any measurable advantage, neither in the mean HR nor in the overall HR. The respondents in the research of JLP found this practice very defendable (1.79 on a scale from 0 to 2). The simulations show that indeed application of ‘Optional Extension’ does not turn experiments with just random data into something significant and the practice does no harm under the condition that the extension is limited to a realistic number of subjects. The practice has also a marginal positive effect on the correlation between sample size and HR.

Each of the QRPs do result in an improvement of the fit. The largest improvement of the fit observed in table sup 1b is produced by the publication bias. Using only the file drawer produces a fit of 9.9, implying that the simulation results and the empirical results do still differ significantly (p = 0.05). Also about 1.6 studies are going into the file drawer for each published study and this figure is larger than the estimate we obtained from the literature (9-11).

### Optimization with Genetic Algorithms

Briefly, GAs work as follows, taking as a simple example the problem of finding the maximum of a real valued function, usually known as the objective function, say *f*(X1,X2), with two integer valued independent variables X1 and X2, and where these variables are constrained to be in the range [0,255]. X1 and X2 can be represented as binary strings of 1’s and 0’s using the Gray code, an alternative to standard binary notation in which successive values differ by only one binary digit or bit. X1 and X2 each require 8 bits and we can concatenate the two 8 bit Gray coded representations of the X’s into a 16 bit vector where the first 8 bits give X1 and second set X2. Such a vector is known as a chromosome in GA parlance and a set of such chromosomes is denoted a generation (all names assigned with a nod to genetics). The GA process starts by producing a set of chromosomes where the bits comprising each chromosome are chosen randomly. In our example we might make 20 chromosomes, each comprised of 16 randomly chosen bits. This is the first generation. The GA algorithm then takes each of these 20 chromosomes, splits them into two 8-bit numbers and converts each of these into base 10 integers. These are X1 and X2 and are evaluated by the objective function *f* to produce a real number. This is repeated for each of the 20 chromosomes. These values of *f* are called fitnesses, because they measure how fit each chromosome is in terms of being close to the sought for maximum. Clearly since the values in the chromosomes in the first generation were chosen randomly the fitnesses that result will range widely and none is likely to be close to the function’s maximum. The algorithm then produces a second generation of chromosomes by combining the chromosomes with the highest fitnesses according to several rules. These include methods for “mating” two chromosomes by splicing them at a random point and combining the head of one with the tail of the other and vice versa, and “mutation” in which single bits of the chromosomes are changed from zero to one or vice versa. As the process is repeated, each generation of chromosomes evolves to represent better fitness values. The chromosome with the highest final fitness gives values for the X’s which meet or very closely approach the function’s maximum. The method can be extended beyond this example to allow for more independent variables and real valued variables. It is also trivial to use the GA method to find the minimum of the objective function, which is the application we use, rather than its maximum,. Further details on GAs are available in the papers referenced (12,13) and are beyond the scope of this paper.

### References

(1) Honorton C. Meta-Analyses of Psi Ganzfeld Research: A response to Hyman. Journal of Parapsychology 1985;49(1):52-86.

(2) Hyman R. The Ganzfeld Psi Experiment: a Critical Appraisal. Journal of Parapsychology 1985;49(1):3-49.

(3) Hyman R, Honorton C. A joint communiqué: The psi ganzfeld controversy. Journal of Parapsychology 1986;50(4):351-364.

(4) Hyman R. Meta-analysis that conceals more than it reveals: Comment on Storm et al.(2010). 2010.

(5) Blackmore S. A report of a visit to Carl Sargent's laboratory. Journal of the Society for Psychical Research 1987.

(6) Hoebens PH. Hello .... do you read me? My adventuere as a parapsychological guinea-pig at the University of Cambridge [Orig. publ. in Dutch in 1980; annotated reprint]. In: Hövelmann GH, Michels JAG, editors. Legitimacy of Unbelief: The Collected Papers of Piet Hein Hoebens. Münster: LiT-Verlag; 2016.

(7) Storm L, Tressoldi PE, Di Risio L. Meta-analysis of free-response studies, 1992–2008: Assessing the noise reduction model in parapsychology. Psychol Bull 2010;136(4):471.

(8) M. Bakker. Good science, Bad science: Questioning Research Practices in Psychological Research, Thesis University of Amsterdam; 2014.

(9) Franco A, Malhotra N, Simonovits G. Social science. Publication bias in the social sciences: unlocking the file drawer. Science 2014 Sep 19;345(6203):1502-1505.

(10) Blackmore S. The extent of selective reporting of ESP Ganzfeld studies. European Journal of Parapsychology 1980;3:213-220.

(11) Watt C. Research Assistants or Budding Scientists. Journal of Parapsychology 2006;70:355-356.

(12) Mebane Jr WR, Sekhon JS. R version of GENetic Optimization Using Derivatives. 2013.

(13) Goldberg DE. E. 1989. Genetic Algorithms in Search, Optimization, and Machine Learning. Reading: Addison-Wesley 1990.
